# Curcumin–Selenium Nanocomposites Integrated into Sol–Gel Siloxane Matrices for Antimicrobial and Delivery Applications

**DOI:** 10.3390/gels12040322

**Published:** 2026-04-10

**Authors:** Florentina Monica Raduly, Valentin Raditoiu, Alina Raditoiu, Iuliana Raut, Adriana Frone, Radu Claudiu Fierascu, Cristian-Andi Nicolae

**Affiliations:** 1 National Institute for Research & Development in Chemistry and Petrochemistry—ICECHIM, 202 Splaiul Independentei, 060021 Bucharest, Romania; monica.raduly@icechim.ro (F.M.R.); coloranti@icechim.ro (A.R.); raut.iuliana@icechim.ro (I.R.); adriana.frone@icechim.ro (A.F.); fierascu.radu@icechim.ro (R.C.F.); ca_nicolae@yahoo.com (C.-A.N.); 2 Faculty of Chemical Engineering and Biotechnology, National University of Science and Technology Politehnica Bucharest, 1–7 Gh. Polizu Street, 011061 Bucharest, Romania

**Keywords:** selenium nanoparticles, curcumin, sol–gel, siloxane matrices, phytosynthesis, controlled release

## Abstract

Selenium nanoparticles (SeNPs) represent promising bioactive agents due to their reduced toxicity and multifunctional biological properties. In this study, SeNPs were synthesized via an eco-friendly phytosynthesis approach using *Curcuma longa* extract, yielding curcumin-functionalized selenium nanoparticles (cur–SeNPs). The composites (cur–SeNPs), either in native extract form or isolated, were incorporated into siloxane hybrid matrices prepared by the sol–gel method from tetraethyl orthosilicate: dimethyldimethoxysilane precursors, with polyvinylpyrrolidone (PVP) as a structural modifier. The host matrices were differentiated by the ratios between the precursors of the siloxane network, 3:1 for CS0–CS4, respectively, 1:1 for CS5, modified with PVP in the case of CS2 and CS3. These were loaded with cur–SeNPs–T in the cases of CS1, CS2, CS5 or with cur–SeNPs for CS3 and CS4. FTIR, XRD, SEM, and EDX analyses confirmed the formation of amorphous siloxane networks with well-dispersed SeNPs (up to ~12 wt%). PVP incorporation generated ordered mesoporous structures, increasing total pore volume sixfold and enlarging the average pore diameter to 9.26 nm. Studies about selenium ion release demonstrate that mesoporosity significantly enhances diffusion-controlled release. Antimicrobial assays against *Staphylococcus aureus*, *Escherichia coli*, and *Candida albicans* reveal a synergistic effect between curcuminoids and SeNPs, particularly in matrices with higher nanoparticle loading. The sol–gel technique for obtaining hybrid materials is very versatile regarding the supports on which the resulting materials or the compounds hosted in these host networks can be deposited. The dynamics of the development of hybrid materials is also reflected in the multitude of applications in various fields such as bio-medical, electronics, agriculture or food. Results obtained in this work highlight the potential of the developed systems for antimicrobial coatings on glass substrates and targeted delivery applications.

## 1. Introduction

Known as one of the essential trace elements for maintaining human, animal, and plant health, selenium—alongside iron, zinc, copper, and others—has been extensively studied over time. Inspired by their crucial role as catalysts in biological processes and their impact on living organisms, researchers have developed new complexes to address the deficiency of these microelements [1,2,3]. Research indicates that the oxidation state of metals significantly influences the cytotoxicity of metal or non–metal ions (M^n+^), whereas their reduced, elemental form (M^0^) generally exhibits lower toxicity. Consequently, the synthesis of metallic and non–metallic nanoparticles has been investigated, leading to the identification and development of new applications due to their unique properties [4,5,6].

Selenium nanoparticles (SeNPs) are synthesized from selenium dioxide (SeO_2_), selenious acid (H_2_SeO_3_), sodium hydrogen selenite (NaHSeO_3_), or sodium selenite (Na_2_SeO_3_) through physical, chemical, or biological methods under varying temperature and pH conditions [7,8,9,10]. Phytosynthesis has recently become a prevalent method, as it is both economical and environmentally friendly. Thus, by utilizing polyphenolic compounds found in plant leaves or roots, ionic selenium is reduced to spherical nanoparticles in most cases, with sizes ranging between 3 and 400 nm, stabilized on the surface by natural compounds with antioxidant effects [11,12,13].

One of the most frequently studied natural compounds for biomedical or eco-friendly applications is curcumin, which is found in the rhizome of turmeric (*Curmuma longa*). It is known as a potent antioxidant, and due to its antifungal, antibacterial, and antiviral properties, it has found applications in various fields such as food additives, active packaging, wound dressings, bioactive textiles, and as an adjuvant in treating cancer, diabetes, and neuronal diseases [14,15,16]. All these applications are limited by the low solubility manifested by curcumin, which has necessitated the development of new conditioning methods through delivery systems such as emulsions, loading into polysaccharides, natural or synthetic polymers, silica, and metallic nanoparticles [17,18,19]. For instance, the curcumin–selenium nanoparticle composite (Cur–SeNP) is designed to address the inherent limitations of its individual components, specifically the poor bioavailability of curcumin and the potential cytotoxicity associated with selenium. By exploiting the synergistic properties of these two compounds, cur-SeNP has demonstrated potential antiviral and bacteriostatic effects [20,21]. Simultaneously, the composite has proven to possess antitumor and chemo–preventive properties, representing a promising aspect for the development of personalized medicine [22,23,24,25]. These applications are largely contingent upon the delivery mechanisms of the active compounds, for which a range of carriers—including polymers, emulsified systems, silica-based materials, peptides, and polysaccharides—have been utilized. Loaded onto various types of mesoporous silica structures, chitosan, or PEG–hyaluronic acid, SeNPs have been tested as antitumor and radioprotective agents. On the other hand, due to their biocidal and antioxidant properties, they have been integrated into wound dressings and diabetic treatments [26,27,28,29,30]. Antimicrobial applications have been extended to active packaging following successful testing against Gram-positive bacteria (*Staphylococcus aureus*, *Bacillus subtilis*, *Bacillus megaterium*), Gram-negative bacteria (*Escherichia coli*, *Klebsiellapneumoniae*, *Pseudomonas aeruginosa*, *Enterobacteraerogenes*), and fungi (*Candida albicans*, *Yarrowialipolytica*, *Saccharomyces cerevisiae*) [12,13,31]. Depending on the intended application, the surfaces of selenium nanoparticles have been designed to be neutral, positively charged, or negatively charged using polyphenolic compounds, polyvinylpyrrolidone (PVP), poly-L-lysine (PLL), and polyacrylic acid (PAA) [31,32,33]. Among these, PVP is most commonly encountered in sol–gel processes where it facilitates the formation of film–forming structures or amorphous silica. In its presence, various models of delivery systems for active compounds have been obtained using sodium alginate, tetraethyl orthosilicate (TEOS), polyvinyl alcohol (PVA), and others, with the aim of achieving high-performance controlled–release systems [34,35,36,37].

The present study aims to synthesize selenium nanoparticles via phytosynthesis using a turmeric extract and subsequently load SeNPs surface-stabilized with curcumin (cur–SeNPs) into siloxane matrices generated through the sol–gel method. This study sets out to develop new host–guest materials and to demonstrate the importance of the network architecture obtained by the sol–gel method with regard to preserving the initial properties of the hosted compounds. Knowing the synergistic effect of curcumin and selenium, it was essential to preserve the antimicrobial properties even after encapsulation in siloxane matrices. At the same time, the objective of this study isto obtain antimicrobial films that would allow deposition on glass substrates that find applications in medical, electronic, smartphone or food-packaging fields. In this manner, coatings with antibacterial properties or targeted delivery systems for cur–SeNP composites were obtained.

## 2. Results and Discussion

It is well established that the turmeric rhizome contains curcumin as its primary constituent, alongside its derivatives, demethoxycurcumin and bisdemethoxycurcumin, respectively. The antioxidant properties of these compounds have been utilized to reduce Se^4+^ ions to Se^0^ nanoparticles, whose surfaces are subsequently stabilized by the curcuminoid derivatives. The resulting composites, either in the form of the natural extract in which they were generated (cur–SeNPs–T) or as selenium nanoparticles isolated from the natural extract (cur–SeNPs), were incorporated into siloxane matrices generated via the sol–gel method. Through this approach, five types of hybrid materials (CS1–CS5) were synthesized, distinguished by the ratios between the siloxane network precursors: tetraethyl orthosilicate (TEOS), dimethyldimethoxysilane (DMDMS), polyvinylpyrrolidone (PVP), and the cur–SeNPs–T or cur–SeNPs composites. Thus, it was possible to study the behavior of the silica host network when loaded with composites with increased hydrophobicity due to the presence of curcumin compounds from the turmeric extract (cur–SeNPs–T) and the influence of these compounds on the architecture of the host network (CS1, CS2 and CS5). In the case of CS2 and CS3, hybrid materials incorporating hydrophobic compounds (CS2) were comparatively evaluated against systems containing compounds of lower hydrophobicity, achieved through the removal of excess curcumin derivatives not bound to selenium nanoparticles (CS3). Concurrently, the influence of precursor composition and their relative ratios on the formation of the host matrix via the sol–gel process under acidic conditions was systematically investigated across the CS0–CS5 series.

### 2.1. Synthesis of the Cur–SeNP Composite in Natural Turmeric Extract

**Figure 1:**
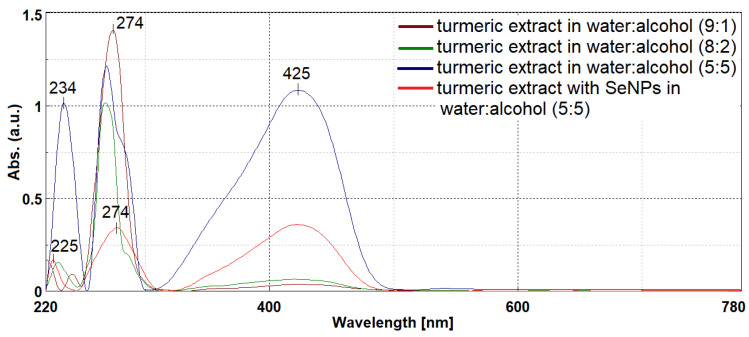
UV-Vis spectrum of the initial aqueous extract with variable ethanol content (10–50%) and the cur–SeNP composite generated in the aqueous turmeric extract.

The cur–SeNP composite was synthesized using a slightly modified method based on Sattar et al. [20], employing an aqueous extract of dried turmeric rhizome and sodium hydrogen selenite (HNaSeO_3_). The formation of selenium nanoparticles was rapidly confirmed by the color transition of the aqueous extract from yellow to reddish–brown upon the addition of HNaSeO_3_. Considering that curcumin derivatives are hydrophobic, in order to increase the yield of natural curcuminoid compounds, variable amounts of ethanol were added. Curcumin derivatives have a very important role in the generation of selenium nanoparticles, as a reducing agent by donating electrons from hydroxyl groups to Se^4+^ ions, transforming them into Se^0^, and then as a stabilizing and anti–caking agent by adhering to the surface of the formed nanoparticles. Therefore, an extract as rich as possible in curcuminoid compounds obtained in the water:ethanol mixture (1:1) was chosen. In the UV–Vis spectra, it is observed that the bands characteristic of the *π*–π* electronic transitions of aromatic systems with absorption maxima at 234 nm and 274 nm have high intensities even in extracts with low alcohol content (Figure 1). This clearly indicates the presence of phenolic compounds and ferulic acid derivatives from turmeric. On the other hand, increasing the proportion of ethanol in the extraction mixture leads to an intensification of the absorption band with a maximum at 425 nm characteristic of curcumin derivatives. After the generation of selenium nanoparticles, the band undergoes a hypochromic effect, which suggests that curcumin molecules participated in the Se^4+^ reduction process. At the same time, it is observed that in the UV range, the band at 234 nm undergoes a hypsochromic shift and a hypochromic effect which is due to the formation of the cur–SeNP complex. The decrease in the intensity of the band at 274 nm together with its broadening confirms the formation of the cur–SeNP complex [11,38].

### 2.2. Obtaining Hybrid Siloxane Materials Loaded with Cur-SeNps

The resulting composite was incorporated into siloxane matrices produced via the sol–gel process. The initial matrix (CS0) was synthesized from a nanosol with a TEOS: DMDMS mass ratio of 3:1. In the case of CS1, the cur–SeNPs–T composite was embedded along with its generation medium, namely the natural turmeric extract. Given the antimicrobial properties associated with curcumin derivatives incorporated into silica networks [16,19] as well as literature reports on the antimicrobial activity of selenium nanoparticles obtained by various methods [7,10,13], it was anticipated that cur–SeNPs–T would exhibit comparable behavior while preserving the individually established properties of its constituent components. However, established studies indicate that the structural model of the host matrix plays a critical role in the behavior of host–guest hybrid materials. To address this, the silica network was modified through the incorporation of PVP as a spacer within the nanosol (CS2). Furthermore, it wasconsidered essential to investigate the behavior of the cur–SeNP composite, isolated from the extract, when embedded within PVP–modified siloxane matrices (CS3).

The CS4 nanosol was synthesized without a spacer and subsequently loaded with cur–SeNPs. Conversely, the CS5 formulation was prepared by increasing the proportion of the DMDMS network modifier to achieve an equimolar ratio with TEOS, followed by loading with cur–SeNPs–T. These hybrid materials were either deposited as films on glass substrates or ground into powders to facilitate the characterization of their structural, optical, and antimicrobial properties.

### 2.3. Morphostructural Characterization of CS0–CS5 Hybrid Materials

**Figure 2:**
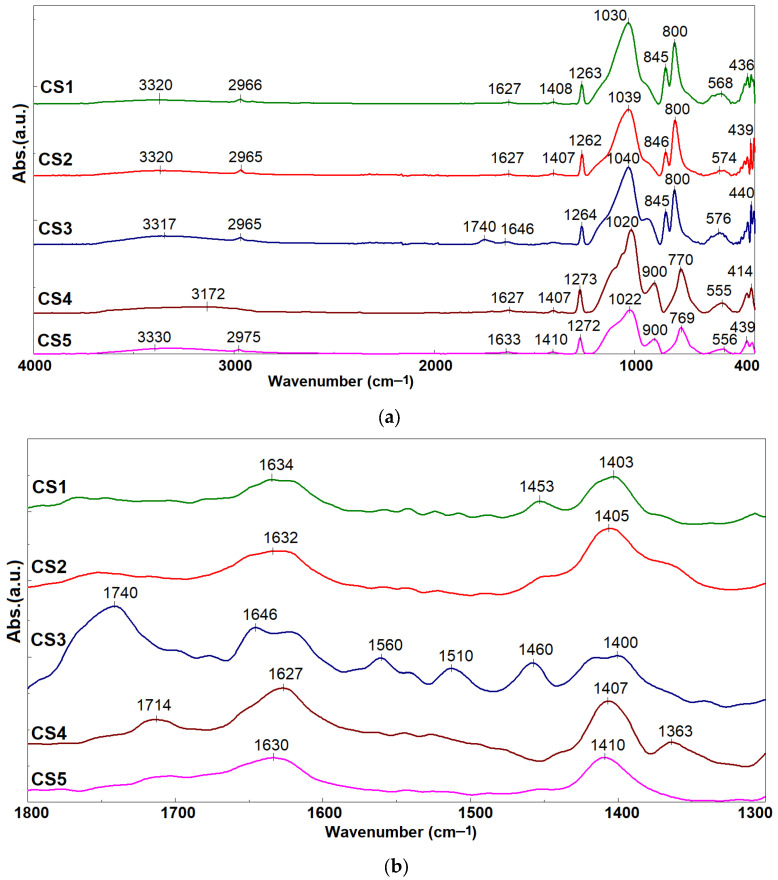
FTIR spectra of hybrid materials CS1–CS5 (**a**) and zoom from 1800 to 1300 cm^−1^ (**b**).

The five synthesized hybrid materials were characterized using FTIR spectra, which exhibited characteristic bands of the siloxane–type host matrix (Figure 2). However, the absorption maxima shifted due to the guest compounds incorporated within these matrices.

The absorption at 3330 cm^−1^ is attributed to –OH groups, present in both the silica networks and the natural curcuminoid compounds. The absorption bands at 1263–1272, 1022–1040, 769–800, and 436–440 cm^−1^ are characteristic of Si–O–Si and Si–O–C stretching vibrations [28,37]. For hybrids CS4 and CS5 that encapsulated a smaller amount of composite, the absorption maximum was recorded at 1272 cm^−1^, while for CS1–CS3 with an increased content of organic compounds, the band characteristic of Si–O–C bond vibrations undergoes a shift with the maximum recorded at 1262 cm^−1^ (CS2) and 1264 cm^−1^ for CS3. The bands in the range 1050–1085 cm^−1^ are characterized by an asymmetrical profile with shifts in absorption maxima resulting from the contributions of C–O bonds, which, in the case of CS1, has a maximum at 1030 cm^−1^. The hybrid materials CS2 and CS3 with PVP content showed a maximum that shifted to 1039 cm^−1^ and 1040 cm^−1^, respectively. The contributions of the C–H and C=C bonds (800–900 cm^−1^) present in the structure of the hosted natural compounds [16] were recorded differently in the case of CS4 and CS5. The band at 900 cm^−1^ is more intense, while the hybrid materials CS1–CS3 with a higher content of embedded composite presented a vibration band with an absorption maximum at 845 cm^−1^. The absorption maximum at 2965 cm^−1^ was assigned to the C–H stretching vibration and it undergoes a shift to 2975 cm^−1^ in the case of CS5 obtained with a higher amount of the DMDMS precursor. The peaks at 1740 and 1646 cm^−1^ (Figure 2b) are characteristic of the C=O stretching bond in PVP [37,39], which is the most prominent in the spectrum of CS3. In the case of CS2, the presence of curcuminoid derivatives in significantly higher quantities resulted in very broad absorption bands with low intensity. The presence of natural compounds is confirmed by the absorption band at 1714 cm^−1^, characteristic of the C=O group in α,β–unsaturated ester compounds [40]. Concurrently, the broad band with a maximum between 1627 and 1630 cm^−1^ (Figure 2b) was attributed to the bending vibrations of O–H groups resulted from adsorbed water molecules [41]. This is further influenced by the interaction between Se atoms and curcuminoid compounds through coordination interactions or surface adsorption, as suggested by Sattar et al. [20], and undergoes a shift in the absorption maximum to 1646 cm^−1^ in the case of CS3.

**Figure 3:**
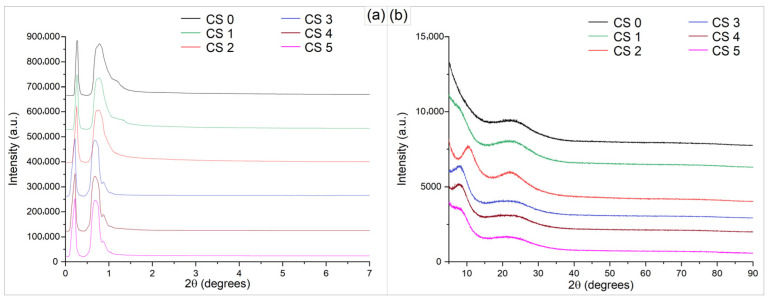
X-ray diffraction patterns at (**a**) low-angle range and (**b**) high-angle range of CS0–CS5.

The structural features of the hybrid silica–based materials (CS0–CS5) were investigated by X–ray diffraction in both low–angle and wide–angle regions, in order to assess the degree of structural organization and the nature of the inorganic network (Figure 3). In the wide–angle region (5–90°), all samples exhibit a broad diffraction halo centered at approximately 20–25°, which is characteristic of amorphous silica. The absence of sharp diffraction peaks indicates that no long–range crystalline order is present within the materials. Furthermore, no reflections associated with crystalline selenium phases are observed. The low–angle diffraction patterns (0–7°) display a single broad maximum at approximately 0.7° (2θ), indicating the formation of weakly organized mesostructures. The noticeable sharpening of the diffraction peak at 2θ = 0.68–0.78° signifies enhanced long–range nanoscale ordering. Simultaneously, the systematic shift toward lower 2θ values from CS0 to CS5 indicates an increase in d–spacing, suggesting progressive structural expansion of the framework, likely driven by compositional–induced rearrangement. The role of precursor ratios (TEOS, DMDMS, and PVP) in shaping the silica network is also reflected in the variation in diffraction profiles across the CS series. High–angle XRD diffractograms confirm the amorphous structure of the host matrix and the silica–based hybrid material, with a broad peak centered at approximately 20.86° (2θ) associated with the (100) plane (ICCD no. 01-087-2096) [42].

**Figure 4:**
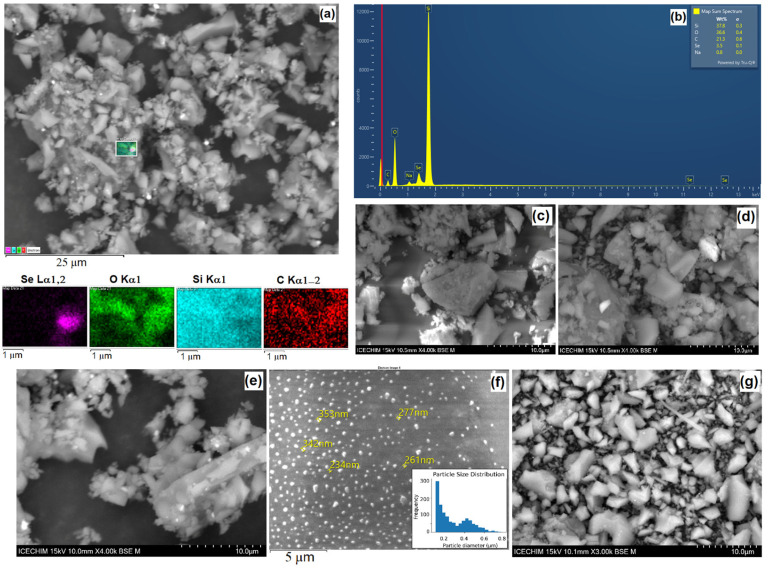
Elemental mapping images used to record the EDX spectrum of CS2 (**a**), EDX spectrum of CS2 (**b**), SEM images of CS3 (**c**), CS0 (**e**), CS1 (**f**), CS5 (**g**) as bulk and CS4 as film (**d**).

The XRD results are supported by the EDX analyses (Figure 4a,b), which recorded the presence of selenium nanoparticles loaded into the silica matrices in varying proportions, as shown in Table 1. It is observed that the percentages of carbon and oxygen vary in relation to the amount of loaded cur–SeNP composite; however, in the case of CS2, the carbon percentage is the highest due to the presence of natural organic compounds from cur–SeNPs–T. On the other hand, the CS3 hybrid material, which was loaded with the largest amount of selenium nanoparticles, exhibits approximately 12% of selenium by mass. Nevertheless, the composition of the hybrid materials is directly influenced by the ratio of the siloxane network precursors, as can be observed by comparing CS1 and CS2. In the case of CS2, the presence of PVP in the nanosol leads to an increase in the percentages of both carbon and silicon compared to CS1, suggesting that the resulting silica network possesses a different structural arrangement through interaction with PVP molecules [37]. These structural modifications lead to the formation of cages within the silica network with more pronounced hydrophilic or hydrophobic properties, which play an important role in the encapsulation process of the guest compounds. The presence of PVP, a polymer with a strong affinity for water (due to polar amide groups) and a less condensed silica network, rich in –OH groups, favors the hydrophilic character. On the other hand, the hydrophobic character appears when the siloxane network (Si-O-Si) is highly crosslinked or when the apolar chain residue of the PVP chain (carbon chain) is oriented towards the outside of the structural “cages”, limiting the access of water. A more hydrophilic network (as in the case of CS2) allows for better stabilization of selenium and curcumin nanoparticles by forming hydrogen bonds between PVP and the active compounds. This prevents the aggregation of nanoparticles and ensures a homogeneous distribution in the host matrix.

In EDX analysis, the K series refers to a specific group of X-rays emitted by atoms in a sample when they are bombarded with an electron beam. When incident electrons (from the electron microscope) strike an atom in the sample, they can eject an electron from the shell closest to the nucleus, called the K shell (energy shell n0 = 1). Within the K series, there are several specific lines, depending on where the electron that fills the hole comes from. For example, in the case of Kα, the electron comes from the next shell (L). This is usually the most intense line in the spectrum. Each chemical element has a unique structure of its electron shells (Figure 4a). Therefore, the energy of the K Series X-rays is specific to each element. The electrostatic force of attraction exerted by the 34 protons of selenium on the electrons in the K shell is immense. To remove an electron from the K shell of selenium, a very high acceleration energy is needed, and an electron falling from the L shell to the K shell releases a large amount of energy (11.2 keV). In contrast, the jump from the M shell to the L shell involves much closer energy levels, resulting in an emission of only 1.3 keV, as can be seen in Figure 4b.

As demonstrated in the SEM images (Figure 4c), the cur–SeNP composite is well dispersed within the host matrix without forming large aggregates. The SEM image of the CS4 nanosol (Figure 4d) deposited on a glass substrate confirms the amorphous phase of the coatings obtained via the sol–gel method. The SiO_2_ particles have a predominantly spherical or pseudo–spherical shape. To generate the particle size frequency histogram, 134 particles were analyzed, with an average diameter of 418 nm ± 112 nm. The histogram analysis shows that the film is moderately polydisperse with minimum particle sizes of 145 nm up to aggregates of 1.15 μm. The calculated Polydispersity Index (PDI) of 0.072 confirms the formation of a near–monodisperse population of particles. This low PDI value suggests a well–controlled synthesis process, where the rate of particle nucleation significantly dominated over the growth and aggregation phases, leading to a narrow size distribution profile. These XRD analysis results (Figure 3b) are further supported by the presence of submicron–sized SiO_2_ particles generated during the hydrolysis process of TEOS.

Curcuminoid compounds are well known to possess a hydrophobic character; consequently, their presence in nanosols influences the structure of the generated silica network. To evaluate these structural effects, the water contact angles of the hybrid material films deposited on glass substrates were measured. The results (Table 2) highlight that the presence of natural compounds leads to a slight increase in the hydrophobic character of the CS1 film compared to the CS0 silica matrix which has the lowest roughness (3.73 nm) and the lowest contact angle (72 degrees), being the most hydrophilic sample. When a small amount of PVP is added to the nanosol, it interacts with the water molecules bound within the network, thereby favoring the encapsulation of more hydrophilic compounds or the formation of hydrogen bonds with the guest compounds. This promotes a structural orientation where hydrophobic groups are directed toward the exterior of the host matrix. This mechanism explains the highest contact angle measured for CS2, followed by CS3, which features an identical host matrix but hosts selenium nanoparticles stabilized on the surface with curcuminoid compounds. At the same time, the increase in roughness from 18.43 nm (CS1) to 25.50 nm and the contact angle to 90 degrees in the case of CS2 demonstrates that the presence of organic components (PVP) “seals” the hydrophilic surface of the silica, increasing the hydrophobic character. Moreover, CS3 presents the highest roughness (29.00 nm), but the contact angle (87 degrees) remains high. This strengthens the hypothesis that selenium nanoparticles and the hybrid matrix create a texture that limits direct contact of water with deep silanol groups. Conversely, the absence of PVP and natural curcuminoid compounds leads to a decrease in the hydrophobicity of the CS4 film. However, increasing the percentage of the DMDMS network modifier affects the structure of the formed silica network, increasing the hydrophobic character of CS5 compared to the CS0 matrix.

From the textural analysis of materials CS0–CS3, it was observed that the hybrid materials possess a low specific surface area—characteristic of film–forming materials—ranging between 0.6 and 3.76 m^2^/g, which is primarily composed of micropores [16]. The total pore volume undergoes modifications upon the introduction of guest compounds into the network, as seen in the CS1 material. In this case, incorporation of the cur–SeNPs composite led to a two–fold increase in the total pore volume, accompanied by a subsequent enlargement of the average pore diameter relative to the CS0 host matrix. Since the guest material is initially introduced into the nanosol, the structure and isomerism of the molecules directly influence the structure of the host matrix network [14]. This effect is particularly pronounced in CS2, where the specific surface area and micropore area exhibit only minor variations relative to the CS0 matrix, whereas the total pore volume increases six–fold. Moreover, the average pore diameter expands to 9.26 nm, compared to 1.81 nm in the silica matrix, and the micropore diameter nearly doubles. In the CS3 hybrid material, the removal of bulky natural compounds leads to a reduction in both the specific surface area and the micropore area, resulting in more homogeneous surfaces with pore diameters substantially smaller than those observed in the CS2 hybrid material.

**Figure 5:**
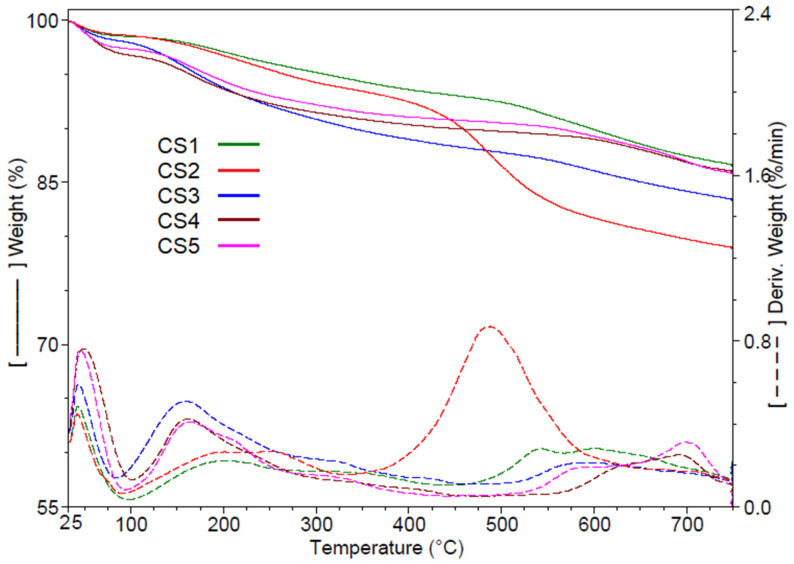
Thermogravimetric curves of hybrid materials CS1–CS5 as bulk.

The thermal stability of the hybrid materials was evaluated through thermogravimetric analysis (TGA) within the temperature range of 25–750 °C (Figure 5). The analysis was conducted under inert conditions to probe the intrinsic thermal stability and decomposition pathways of the silica–based hybrid materials in the absence of oxidative interference. Such an approach suppresses the combustion of the organic constituents (PVP and curcumin derivatives) and prevents potential oxidation of SeNPs, thereby enabling a more reliable discrimination of the successive mass–loss events. Based on the data presented in Table 3, all hybrid materials exhibit a mass loss between 1.36% and 3.25% up to 100 °C, which is most likely attributed to the evaporation of surface–adsorbed water molecules. The subsequent decomposition stage occurs between 100 and 480 °C and is assigned to thermal processes associated with the loss of interstitial water. In sol–gel chemistry, water is both a reactant and a by–product. As discussed, the presence or absence of PVP plays an important role in the formation of “cages” in the silica network and acts as reservoirs for sequestering interstitial water, which is released upon heating. This phenomenon is crucial because the release of interstitial water precedes the thermal degradation of the organic components (PVP/curcumin derivatives), which occurs at temperatures above 250 °C. A reduction in the loading percentage of the composites within the host matrix promotes an increase in the proportion of water retained in the network, with the corresponding thermal release occurring at an earlier stage, at approximately 160 °C. Accordingly, CS1 and CS2, which possess relatively similar pore surface areas, demonstrate comparable behavior, losing 5.4% and 4.9% of their mass, respectively, around 200 °C. A similar trend is observed for CS4 and CS5, which exhibit mass losses of 6.9% and 6.6%, respectively, at 160 °C. The CS3 composite exhibits the most pronounced mass loss within this temperature interval (10.10% at 160 °C). This behavior is likely attributable to its film–forming structure, in which a fraction of the guest compounds located near the surface is released without the requirement for additional thermal energy input. In the final stage, ranging from 480 to 750 °C, the decomposition of aromatic residues takes place. The most significant mass loss (14.7%) was recorded for CS2, the hybrid material with the highest loading of cur–SeNPs–T. This occurred at 487.5 °C, a temperature corresponding to the degradation of aromatic fragments belonging to curcumin derivatives [19]. The remaining materials exhibit mass losses ranging from 3.8% to 6.5% at temperatures between 541 °C (for CS1, containing cur–SeNPs–T) and 623 °C (for CS4, characterized by a lower organic content and a more stable silica network). In the cases of CS4 and CS5, which possess the most stable silica networks, a final decomposition step is observed at approximately 700 °C, corresponding to the collapse of the silica framework. At the completion of the thermal decomposition process, all samples yield a residual mass between 83% and 86%, attributed to the formation of inorganic silicate–type material.

### 2.4. Optical Properties of the CS0–CS5 Hybrid Materials

**Figure 6:**
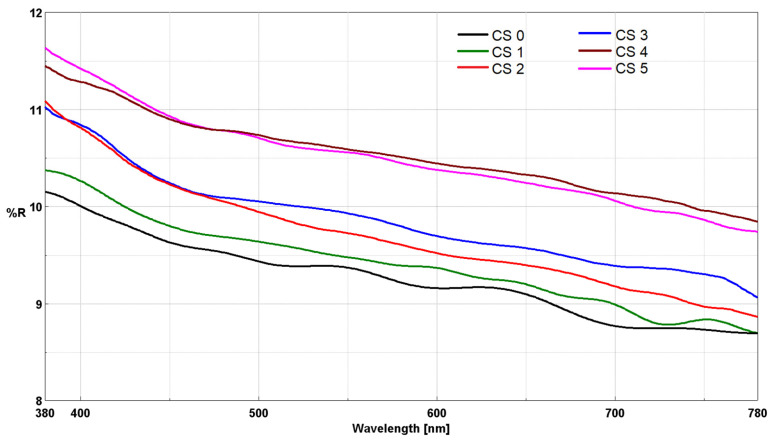
UV-Vis reflectance of CS0–CS5 films obtained by the sol–gel method and deposited on glass support.

The hybrid materials were deposited as thin films on glass substrates. The optical properties of the film–forming materials, in relation to the nanosol composition, were evaluated by measuring reflectance (Figure 6) in the UV-Vis range. The recorded reflectance spectra show continuous and smooth curves, without “noise” or sudden fluctuations, which indicates a homogeneous surface and the absence of major structural defects that could cause light scattering.

The results for reflectance measured at 550 nm provide relevant information regarding the optical properties and surface characteristics of the synthesized materials, which are summarized in Table 4. The values represent the average of three measurements taken at different points of each sample, with a standard deviation below <0.5%, thus confirming the uniformity of the deposition over the entire glass surface. Based on the recorded values, it is observed that the reflectance of the films is lower (CS0) compared to that of the glass substrate (R_550_ = 10.3%) [19]. Reflectance increases slightly in the presence of the cur–SeNPs–T composite in CS1, CS2, and CS5, likely due to the intermolecular hydrogen bonds established between the host matrix and the functional groups of the natural compounds. Conversely, upon removal of the natural compounds from the CS3 and CS4 nanosols, a more pronounced increase in reflectance is observed. This enhancement can be attributed to the intrinsic optical properties of the selenium nanoparticles, which are strongly dependent on their size and morphology [43,44], as well as to the intermolecular hydrogen bonding established between the curcuminoid derivatives—responsible for stabilizing the nanoparticle surface—and the host matrix [16].

In the case of hybrid materials with a high content of natural compounds, namely CS1 and CS2, a decrease in luminosity was observed (L* = 80.96 for CS2), accompanied by a shift toward yellow with b* values nearly doubling. The high content of selenium nanoparticles loaded into CS3, combined with a lower presence of natural compounds in the matrix, leads to a reduced number of intramolecular bonds. Consequently, for CS3, the luminosity increases (L* = 90.64), and the hue shifts toward red, following an increase in a* = 10.11. The hybrid materials CS4 and CS5 further confirm this theory; in these cases, the low content of natural compounds reduces the interactions between the host matrix and the guest composite, resulting in bright materials with slight shifts toward yellow, evidenced by b* values of 5.68 (CS4) and 7.03 (CS5), respectively.

### 2.5. Evaluation of Selenium Ion Release Potential

**Figure 7:**
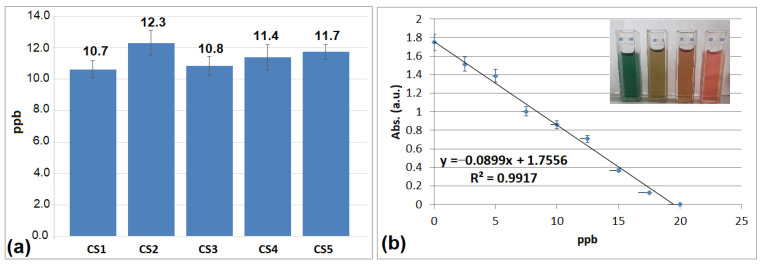
The concentration of Se ions in the aqueous solution and standard error after one hour of suspension in water of hybrid materials C1–C5 (**a**) and calibration graph of DTZ standard solution (0.48 mM) with increasing amounts of Se ions (0–25 ppb) (**b**). The data presented graphically represent the average of three measurements performed for each sample.

Given that the hydrolysis of TEOS can generate silica with varying degrees of porosity depending on synthesis conditions (acidic or basic media) [45,46,47], tests were conducted to evaluate the release potential of selenium ions from the host matrix (Figure 7a). The assay was performed according to a method previously published by our group [48], utilizing a dithizone (DTZ) solution to interact with the selenium ions released from the silica framework. The efficacy of the method is rapidly confirmed by the color transition of the DTZ solution from green to reddish–brown upon complexation with selenium ions (Figure 7b). The complexation of selenium ions was assessed by spectrophotometric analysis at 620 nm, with concentrations determined from a calibration curve constructed using a 100 mM HNaSeO_3_ standard solution.

Samples were measured after one hour of dispersion in water. The results demonstrate that all investigated silica–based delivery systems possess the capacity to release selenium ions. Among these, CS2 exhibited the highest performance, as anticipated, given its superior total pore volume and largest average pore diameter according to the BET analysis results (Table 2). Consequently, the mesoporous network of CS2 facilitates a much easier release of selenium ions. Due to its structural characteristics, where even the micropores possess relatively large dimensions (2.9 nm) compared to the other studied delivery systems (1.54 nm), water can readily access the hosted nanoparticles. In comparison, CS1 and CS3 show similar performance regarding selenium ion release; although the pore volume and average diameter are structurally higher in CS3, the micropore diameters are identical. Thus, their size limits water access to the nanoparticles, thereby reducing the ion release capacity from the matrix.

### 2.6. Antimicrobial Properties of the CS1–CS3 Hybrid Materials

Selenium is an essential trace element for human health [1,5,7], being a cofactor for enzymes such as glutathione peroxidase, which protect cells against oxidative stress. Its incorporation in the form of nanoparticles allows for a controlled release that can support local antioxidant homeostasis. Curcumin acts as a natural stabilizing agent (capping agent) for nanoparticles but also brings its own antioxidant and anti–inflammatory profile [14,17], enhancing the biosafety of the material. From a biological point of view, the combination of the two active compounds and the use of the cur–SeNPs–T system offer major advantages over conventional biocides. Therefore, the developed host–guest system functions as a ‘bio-active’ material: while the silica network restricts the proliferation of pathogens, the encapsulated components (Se and curcumin derivatives) can contribute to reducing oxidative stress at the interface with living tissue, transforming the coating into a biocompatible protective barrier. To elucidate further the mechanisms and performance of the controlled–release delivery systems under study, the three hybrid materials (CS1, CS2, and CS3) were evaluated for their antimicrobial activity against the bacteria *E. coli* and *S. aureus*, and the fungus *C. albicans*. The results, recorded 24 h after the application of the hybrid materials onto the microbial cultures, are summarized in Table 5. Given the established antimicrobial properties of both selenium [12,13] and curcuminoid derivatives [14,19], CS1 and CS2 exhibited, as anticipated, similar antimicrobial activity. The inhibition zone values reflect the synergistic effect of the two types of structures within the cur–SeNPs–T composite.

Interestingly, in the case of CS3, the concentration of natural compounds is significantly reduced; nevertheless, it demonstrated higher antimicrobial activity compared to CS1 and CS2. These results suggest that the concentration of selenium nanoparticles is lower in the CS1 and CS2 delivery systems, thereby reducing and limiting the release of selenium ions over time. It was also found that, although CS2 has a much larger total pore volume (0.0035 cm^3^/g) compared to 0.0018 cm^3^/g (CS1) and an average pore diameter of 9.26 nm, both have similar antimicrobial activity (18 and 20.5 mm). This more open structure compensates for the fact that it has a larger contact angle (90 degrees), allowing for efficient diffusion of ions through the network channels. However, in the case of CS3, even though the silica network structure has a lower S_BET_–specific surface area (0.63 m^2^/g), the pore volume remains high (0.0032 cm^3^/g) so that the high concentration of the cur–SeNPs composite ensures a prolonged and continuous release of selenium ions. This indicates that the network is not blocked but contains large “reservoirs” of the cur–SeNPs composite that continuously supply the surface with antimicrobial agents. Thus, the concentration of the guest compound within the host matrix plays a pivotal role in the manifestation of antimicrobial properties in host–guest hybrid systems. The antimicrobial activity of the hybrid materials (Table 5) is considered remarkable, especially for the CS3 sample, which equals the performance of Clindamycin against *S. aureus* and significantly exceeds the efficiency of Clotrimazole in the case of *C. albicans*. These results are directly correlated to the high concentration of the cur–SeNPs composite and the ability of the silica network to ensure a sustained release of selenium ions, so that their efficiency is superior compared to the data in the specialized literature [49,50]. The use of selenium and curcumin, both recognized for their antioxidant properties and beneficial role in human metabolism, gives these film–forming coatings a ‘bio-active’ character. Thus, the resulting hybrid materials not only eliminate pathogens, but also actively contribute to the antioxidant protection of the surface, representing a sustainable and biocompatible alternative to conventional antimicrobial agents.

## 3. Conclusions

The present study successfully demonstrates the synthesis of selenium nanoparticles (SeNPs) through an eco–friendly phytosynthesis method using a turmeric (*Curcuma longa*) extract. The resulting cur–SeNP composites were effectively incorporated into hybrid siloxane matrices via the sol–gel process, utilizing TEOS and DMDMS as precursors and PVP as a structural modifier. Morphostructural characterization through FTIR, SEM and XRD confirms the formation of amorphous siloxane networks, where selenium nanoparticles were well–dispersed without significant aggregation. EDX analysis validates that the selenium loading reached up to 12% by weight in the optimized hybrid materials. The structural modification of the silica network by PVP (the CS2 and CS3 series) proves essential in tailoring the hydrophobicity and porosity of the matrices. Specifically, the addition of PVP led to a sixfold increase in the total pore volume and a significant expansion of the average pore diameter (up to 9.26 nm), facilitating a more efficient encapsulation of guest compounds. Optical investigations reveals that the reflectance properties were directly influenced by the size of the nanoparticles and the intermolecular hydrogen bonds between the curcuminoid derivatives and the host matrix. Furthermore, colorimetric analysis in the CIEL*a*b* system shows that these materials maintain high luminosity (L* > 80), with hue shifts toward yellow or red depending on the concentration of natural extracts and SeNPs. Evaluation of selenium ion release and antimicrobial tests demonstrate that the mesoporous structure of the host matrix plays a decisive role in the performance of the active compound release process. These hybrid materials represent a promising platform for the development of bioactive coatings or targeted delivery systems, with potential applications in personalized medicine, antimicrobial packaging, and wound dressings.

## 4. Materials and Methods

### 4.1. Obtaining the Mixture of Hybrid Materials

The aqueous extract of *Curcuma longa* was prepared from 2 g of turmeric powder which was mixed with 200 mL of deionized water with variable ethanol content (10–50%) and heated at 65 °C for 2 h with continuous magnetic stirring to facilitate the extraction of curcuminoids, flavonoids and phenolic compounds. The resulting mixture was first filtered and then centrifuged at 8000 rpm for 15 min to remove any remaining particles. The resulting clear yellow solution was continuously used as a reducing agent and stabilizer on the surface of the generated selenium nanoparticles. For the phytosynthesis of selenium nanoparticles, 50 mL of 0.01 M sodium selenite solution (HNaSeO_3_) was mixed with 50 mL of turmeric extract, at pH = 8, adjusted with ascorbic acid (0.05 M). The mixture obtained was stirred continuously for 4 h at 55 °C, following the change in the color of the solution from yellow to brick–red. The color change was the first indication that selenium nanoparticles were formed. The resulting solution was divided into two, half was used as such (cur–SeNP–T) and the other was separated by centrifugation at 10,000 rpm, for 15 min. Isolated SeNps were suspended in ethyl alcohol, resulting in a 0.002% concentration solution (cur–SeNP), from which it was embedded in the CS3 and CS4 matrix.

The silica–based matrices in which the composites (cur–SeNP–T) and (cur–SeNP) were loaded were synthesized by the sol–gel method, using TEOS:DMDMS as network precursors, 3:1 (*v*/*v*) for the hybrid materials C0–C4 and 1:1 (*v*/*v*) for CS5. The gels were generated in the presence of 0.1 N nitric acid, under magnetic stirring for 6 h at room temperature. For CS2 and CS3, the silica networks were modified with PVP (0.05% concentration). All siloxane matrices were loaded with the composite (cur–SeNP–T) or (cur–SeNP), as indicated in Table 6. The nanosols were deposited onto microscope glass slides using the doctor blade technique. Prior to deposition, the substrates were cleaned sequentially with a 30% hydrogen peroxide solution and acetone, followed by drying. The resulting films were dried at room temperature for 24 h and subsequently thermally treated in an oven at 110 °C for 4 h. The residual nanosol remaining after film deposition was allowed to undergo solvent evaporation at room temperature for 24 h, followed by heat treatment at 110 °C for 4 h. The resulting solid materials were ground using a mortar and pestle, and the obtained powders were stored in sealed containers at room temperature. All reagents used in this work were obtained from Aldrich, St. Louis, MO, USA and were used without further purification.

### 4.2. Characterization Methods

#### 4.2.1. Fourier–Transform Infrared (FTIR) Spectroscopy

The CS0–CS5 hybrid materials in powder form were analyzed by FTIR measurements, recorded with a JASCO FT–IR 6300 instrument (Jasco Int. Co., Ltd., Tokyo, Japan), equipped with a Specac ATR Golden Gate (Specac Ltd., Orpington, UK) with KRS5 lens, in the range of 400 to 4000 cm^−1^ (32 accumulations at a resolution of 4 cm^−1^).

#### 4.2.2. Thermogravimetric Analysis (TGA)

Their thermal stability was evaluated by TGA using a TGA Q5000IR instrument (TA Instruments, New Castle, DE, USA). The 14.7–15.4 mg samples were analyzed in platinum pans under the following conditions: ramp 10 °C/min to 750 °C, isothermal for 5 min, two gases (purge gas 1: Nitrogen 5.0 (99.999%), 50 mL/min and purge gas 2: Synthetic Air 5.0 (99.999%), 50 mL/min).

#### 4.2.3. Porosimetric Surface Characterization

Nova 2200e automated gas adsorption system Quantachrome (Quantachrome Instruments Corporate Drive, Boynton Beach, FL, USA) was used to characterize the textural properties of CS0–CS3. The specific surface areas (S_BET_) and total pore volumes (V_total_) of the samples degassed at 130 °C for 4 h were determined from the N_2_ adsorption–desorption isotherms measured at −196 °C. The specific surface area of the micropores was estimated using the Dubinin–Radushkevich (DR) method obtained from low–pressure gas adsorption isotherms. The micropore size (pores with diameter < 2 nm) was calculated using the DFT (Density Functional Theory) method using the slit pore model.

#### 4.2.4. X–Ray Diffraction (XRD) Analysis

X–ray diffraction (Rigaku Corporation, Tokyo, Japan) analyses were performed with a RigakuSmartLab diffactometer, having the following operating conditions: 45 kV, 200 mA, Cu Kα_ radiation parallel beam configuration (2θ/θ_ scan mode), from 0.02 to 90 2θ_ degrees. The diffractograms were interpreted using the Rigaku Data Analysis Software PDXL 2 (ver. 2.7.2.0, Rigaku Corporation, Tokyo, Japan), by comparison with ICDD (International Center for Diffraction Data) database entries.

#### 4.2.5. Scanning Electron Microscopy (SEM) Analysis

SEM images were obtained with a TM4000Plus equipment (HITACHI, Tokyo, Japan) using the following operating conditions: acceleration voltage of 15 kV and magnifications up to 1800× and an energy–dispersive X–ray spectrometer (EDS, model X–stream–2 from Oxford Instruments, Oxford, UK) in the SEM configuration enabling to analyze the elemental composition using AZtecOne 1.0 software from Oxford Instruments. The data for generating the particle size distribution graph were processed using a free software, ImageJ (ImageJ, https://imagej.net/ij/, accessed on 20 March 2026), by processing the BSE (Backscattered Electrons) image.

#### 4.2.6. Measuring the Contact Angle of Films

The hydrophilic properties of the coatings on glass support were measured in air, at 25° C and ambient humidity, 2 s, by measuring the water contact angle with a CAM 200 (KSV Instruments, Helsinki, Finland) equipped with a high–resolution camera (Basler A602f, Basler, Ahrensburg, Germany) and an auto dispenser. Drops of 6 µL deionized water were dispensed on each film, and the value of the reported water contact angles was the average of six measurements.

#### 4.2.7. Film Roughness Measurement

The root mean square roughness (Rq) of the hybrid materials was measured using NanoScope AFM software (version 1.20, MultiMode 8 microscope, Bruker, Santa Barbara, CA, USA) on the basis of unprocessed topographic AFM images of 5 × 5 μm^2^.

#### 4.2.8. Reflectance Spectra and Color Measurements

The diffuse reflectance spectra of the hybrid films CS1–CS5 were recorded on a JASCO V570 UV–VIS–NIR spectrophotometer (Jasco Int. Co., Ltd., Tokyo, Japan), equipped with a JASCO ILN–472 (150 mm) integrating sphere (Jasco Int. Co., Ltd., Tokyo, Japan), using uncovered support as reference. The same apparatus was used to measure the total color differences in the CIELAB system using a 10° standard observer and illuminant D65, using spectralon as the reference for the hybrid materials in powder form.

#### 4.2.9. Antibacterial Activity

The antibacterial activity of the hybrid materials CS1–CS3 against bacterial strains *Staphylococcus aureus* (ATCC 25923), *Escherichia coli* (ATCC 25922) and the fungal strain *Candida albicans* (ATCC 10231) was evaluated by the diffusion method. The method was performed in Petri dishes on specific agar media: Mueller Hinton agar for the bacterial strains *E. coli*, and *S. aureus*, and Sabouraud agar for the fungal strain *C. albicans*. The working inoculum was a standardized suspension prepared from a fresh 18–24 h microbial culture. This suspension was adjusted in sterile physiological saline (AFS) to a density of 1–3 × 10^8^ CFU/mL, which is equivalent to the 0.5 McFarland standard. Density was further adjusted spectrophotometrically by measuring the absorbance at 600 nm to achieve an optimal optical density. All samples were inoculated aseptically at a volume of 30 μL per spot onto the agar medium. For bacterial strains, the inoculated Petri dishes were incubated for 24 h at 37 °C. For the fungal strain *C. albicans*, plates were incubated at 28 °C. Antimicrobial activity was assessed by measuring the diameter of the zone of inhibition (halo) that appeared around the hybrid materials.

## Figures and Tables

**Table 1:** Percentage weight of elements in CS0–CS5 using SEM-EDX.

Element	Line Type	CS0	CS1	CS2	CS3	CS4	CS5
O	K series	45.9 ± 1.1	28.1 ± 0.7	36.6 ± 0.4	29.3 ± 0.5	30.9 ± 0.5	37.5 ± 0.5
C	K series	23.9 ± 1.6	38.0 ± 1.2	21.3 ± 0.6	13.6 ± 0.2	22.2 ± 0.7	23.9 ± 0.9
Se	L series	–	2.2 ± 0.2	3.5 ± 0.1	12.1 ± 0.2	6.7 ± 0.2	6.9 ± 0.2
Si	K series	27.3 ± 0.6	15.8 ± 0.4	37.8 ± 0.3	30.5 ± 0.0	26.7 ± 0.2	25.5 ± 0.3
Na	K series	1.4 ± 0.1	0.9 ± 0.1	0.8 ± 0.0	4.7 ± 0.1	0.4 ± 0.1	5.2 ± 0.1
Cu	L series	–	4.0 ± 0.3	–	9.8 ± 0.4	12.3 ± 0.3	0.3 ± 0.2

**Table 2:** Contact angle and square roughness for CS0–CS5 films and porosimetric properties of CS0–CS3 powders.

Sample	Contact Angle (Degrees)	Square Roughness (nm)	S_BET_ (m^2^/g)	S_DRmicropore_ (m^2^/g)	V_total_ (cm^3^/g)	D_med_ (nm)	D_poreDFT_ (nm)
CS0	72 ± 2.6	3.73 ± 1.2	1.55	1.27	0.0007	1.81	1.54
CS1	79 ± 1.4	18.43 ± 0.4	3.76	1.29	0.0018	1.92	1.54
CS2	90 ± 2.5	25.50 ± 1.7	1.80	1.17	0.0035	9.26	2.90
CS3	87 ± 1.5	29.00 ± 0.8	0.63	0.00011	0.0032	4.75	1.54
CS4	75 ± 1.6	18.74 ± 0.5	–	–	–	–	–
CS5	84 ± 1.2	17.78 ± 0.6	–	–	–	–	–

S_BET_—specific surface area of hybrid materials using Brunauer–Emmett–Teller method; S_DRmicropore_—specific surface area of micropore using Dubinin–Radushkevich method; V_total_—total pore volume; D_med_—the average pore size; D_poreDFT_—determination of pore size by Density Functional Theory method.

**Table 3:** Characteristic decomposition temperatures and mass losses for CS1–CS5.

Sample	RT–100 °C	100–480 °C		480–750 °C		Residue at 750 °C	
	Wt. Loss (%)	Wt. Loss (%)	T_max_ (°C)	Wt. Loss (%)	T_max_ (°C)	N_2_ (%)	Air (%)
CS1	1.49	5.40	207.2	6.46	543.2	86.65	86.42
CS2	1.36	4.94	201.6	14.70	487.5	79.00	78.66
CS3	1.85	10.10	159.7	4.59	591.7	83.46	83.21
CS4	3.25	6.91	161.8	3.78	622.8	86.06	85.97
CS5	2.61	6.61	163.5	4.90	593.3	85.88	85.75

**Table 4:** UV-Vis reflectance at 550 nm for the films deposited on glass support and CIEL*a*b* color space for their powders.

Materials	R_550_ (%)	L*	a*	b*
CS0	9.4	94.19	−0.99	5.71
CS1	9.5	84.46	6.49	17.19
CS2	9.7	80.96	10.11	14.07
CS3	9.9	90.64	1.41	13.75
CS4	10.6	94.06	−1.03	5.68
CS5	9.5	93.72	0.05	7.03

The color parameters for the five types of hybrid materials were measured using the powder form obtained after grinding. Measurements in the CIELab* system demonstrated, as previously highlighted in other studies [16], that the silica matrix modified with methyl groups from DMDMS leads to the production of bright hybrid materials with a luminosity L* > 80 and a hue shift toward yellow, indicated by b* > 5.5, due to the hydrogen bonds established between the host matrix and the guest composite.

**Table 5:** Antimicrobial activities (zone of inhibition in mm) of CS1–CS3.

Sample	*Staphylococcus aureus*	*Escherichia coli*	*Candida albicans*
Zones of inhibition	(mm)	(mm)	(mm)
CS1	19.5 ± 0.5	19.0 ± 0.9	20.0 ± 1.0
CS2	19.5 ± 0.7	18.0 ± 1.0	20.5 ± 1.5
CS3	23.0 ± 0.5	25.0 ± 0.8	30.0 ± 1.2
Clindamycin (2 μg)	23.0 ± 0.5	–	–
Norfloxacin (10 μg)	–	31.0 ± 0.8	–
Clotrimazole (10 μg)	–	–	14.0 ± 0.9

The tests for the evaluation of antimicrobial activity for hybrid materials C1–C3 were performed in triplicate. The data presented in Table 5 represent the average of the three determinations for each sample.

**Table 6:** The composition of the obtained nanosols C0–C5.

Materials	TEOS [mL]	DMDMS [mL]	THF [mL]	HNO_3_ [mL]	PVP [mL]	Cur–SeNps–T [mL]
CS0	2.4	0.8	3	0.1	–	–
CS1	2.4	0.8	3	0.1	–	2.2
CS2	2.4	0.8	3	0.1	0.1	2.2
CS3	2.4	0.8	4	0.1	0.1	1.2 *
CS4	2.4	0.8	4.6	0.1	–	0.6 *
CS5	1.6	1.6	3	0.1	–	0.6

* alcoholic solution 0.002% SeNps isolated from turmeric extract (Cur–SeNps).

## Data Availability

Data are contained within the article.

## References

[1] Zazzo J.-F. (1993). Oligo-éléments, vitamines et immunité. Nutr. Clin. Métab..

[2] Tiekink E.R.T. (2021). Supramolecular aggregation patterns featuring Se⋯N secondary bonding interactions in mono-nuclear selenium compounds: A comparison with their congeners. Coord. Chem. Rev..

[3] Smethurst D.G.J., Shcherbik N. (2021). Interchangeable utilization of metals: New perspectives on the impacts of metal ions employed in ancient and extant biomolecules. J. Biol. Chem..

[4] Singh J., Dutta T., Kim K.-H., Rawat M., Samddar P., Kumar P. (2018). ‘Green’ synthesis of metals and their oxide nanoparticles: Applications for environmental remediation. J. Nanobiotechnol..

[5] Carvalho-Silva J.M., dos Reis A.C. (2025). Antiviral activity of silver and selenium nanoparticles against SARS-CoV-2: A comprehensive systematic review of in vitro, in vivo, and clinical evidence. J. Trace Elem. Med. Biol..

[6] Vennela A.B., Senthilkumar N., Hemalatha K.V. (2025). Neodymium-doped Co_3_O_4_ nanoparticles prepared via PVP-assisted Sol-gel method for improved visible-light photocatalytic activity. Mater. Sci. Eng. B.

[7] Pandey S., Awasthee N., Shekher A., Rai L.C., Gupta S.C., Dubey S.K. (2021). Biogenic synthesis and characterization of selenium nanoparticles and their applications with special reference to antibacterial, antioxidant, anticancer and photocatalytic activity. Bioprocess Biosyst. Eng..

[8] Piacenza E., Presentato A., Ferrante F., Cavallaro G., Alduina R., Chillura Martino D.F. (2021). Biogenic Selenium Nanoparticles: A Fine Characterization to Unveil Their Thermodynamic Stability. Nanomaterials.

[9] Dwivedi C., Shah C.P., Singh K., Kumar M., Bajaj P.N. (2011). An Organic Acid-induced Synthesis and Characterization of Selenium Nanoparticles. J. Nanotechnol..

[10] Geng L., Li L., Sun X., Cheng S., He J. (2025). Recent Advances Towards Selenium Nanoparticles: Synthetic Methods, Functional Mechanisms, and Biological Applications. Foods.

[11] Saravanan K., Madhaiyan M., Periyasamy P., Manivannan P., Bayrakdar A., Balakrishnan V. (2025). Green synthesis and detailed characterization of selenium nanoparticles derived from *Alangium salviifolium* (L.f) Wangerin. Chem. Phys. Impact.

[12] Hussain A., Lakhan M.N., Hanan A., Soomro I.A., Ahmed M., Bibi F., Zehra I. (2023). Recent progress on green synthesis of selenium nanoparticles—A review. Mater. Today Sustain..

[13] Odemis O., Alan Y., Agırtas M.S. (2025). Plant-mediated synthesis of selenium nanoparticles via *Juglans regia* and *Mentha piperita*: A dual-source approach for antimicrobial applications. Nano-Struct. Nano-Objects.

[14] Raduly F.M., Raditoiu V., Raditoiu A., Purcar V. (2021). Curcumin: Modern Applications for a Versatile Additive. Coatings.

[15] Deng X., Ratnayake J., Ali A. (2025). Curcumin-Loaded Drug Delivery Systems for Acute and Chronic Wound Management: A Review. Bioengineering.

[16] Raduly F.M., Rădițoiu V., Rădițoiu A., Frone A.N., Nicolae C.A., Răut I., Constantin M., Grapin M. (2023). Multifunctional Finishing of Cotton Fabric with Curcumin Derivatives Coatings Obtained by Sol–Gel Method. Gels.

[17] Alshammari E.M. (2025). Curcumin-based biocompatible nanocarriers: A contemporary perspective in functional foods and biomedical applications. Discov. Nano.

[18] Zheng B., McClements D.J. (2020). Formulation of More Efficacious Curcumin Delivery Systems Using Colloid Science: Enhanced Solubility, Stability, and Bioavailability. Molecules.

[19] Raduly F.M., Rădițoiu V., Rădițoiu A., Frone A.N., Nicolae C.A., Purcar V., Ispas G., Constantin M., Răut I. (2022). Modeling the Properties of Curcumin Derivatives in Relation to the Architecture of the Siloxane Host Matrices. Materials.

[20] Sattar Z.A., Mohammed A.M., Khalaf Y.H., Eisa M.H., Ramizy A. (2025). Selenium nanospheres: Synthesis and evaluation as a potential anticancer, antimicrobial, and antioxidant agents. Mater. Chem. Phys..

[21] Huang Y., Su E., Ren J., Qu X. (2021). The recent biological applications of selenium-based nanomaterials. Nano Today.

[22] DavaraniAsl F., MohammadiArvejeh P., Rezaee M., Saffari-Chaleshtori J., Deris F., Satari A., Asgharzadeh S., Khosravian P. (2025). Folic Acid Targeted Selenium–Curcumin Nanoparticles to Enhance Apoptosis in Breast Cancer Cells. Chem. Biodivers..

[23] Guo M., Li Y., Lin Z., Zhao M., Xiao M., Wang C., Xu T., Xia Y., Zhu B. (2017). Surface decoration of selenium nanoparticles with curcumin induced HepG2 cell apoptosis through ROS mediated p53 and AKT signaling pathways. RSC Adv..

[24] Yu S., Wang Y., Zhang W., Zhang Y., Zhu W., Liu Y., Zhang D., Wang J. (2016). pH-Assisted surface functionalization of selenium nanoparticles with curcumin to achieve enhanced cancer chemopreventive activity. RSC Adv..

[25] Ghoflchi S., Karav S., Hosseini H., Sahebkar A. (2026). Curcumin-selenium nanoparticles: A promising approach in disease prevention and treatment. J. Trace Elem. Med. Biol..

[26] Kumari M., Purohit M.P., Patnaik S., Shukla Y., Kumar P., Gupta K.C. (2018). Curcumin loaded selenium nanoparticles synergize the anticancer potential of doxorubicin contained in self-assembled, cell receptor targeted nanoparticles. Eur. J. Pharm. Biopharm..

[27] Fang M., Zhang H., Wang Y., Zhang H., Zhang D., Xu P. (2023). Biomimetic selenium nanosystems for infectious wound healing. Eng. Regen..

[28] Wang M., Sun X., Wang Y., Deng X., Miao J., Zhao D., Sun K., Li M., Wang X., Sun W. (2022). Construction of Selenium Nanoparticle-Loaded Mesoporous Silica Nanoparticles with Potential Antioxidant and Antitumor Activities as a Selenium Supplement. ACS Omega.

[29] Zhang H., Sun Q., Tong L., Hao Y., Yu T. (2018). Synergistic combination of PEGylated selenium nanoparticles and X-ray induced radiotherapy for enhanced anticancer effect in human lung carcinoma. Biomed. Pharmacother..

[30] Karthik K.K., Cheriyan B.V., Rajeshkumar S., Gopalakrishnan M. (2024). A review on selenium nanoparticles and their biomedical applications. Biomed. Technol..

[31] Galić E., Ilić K., Hartl S., Tetyczka C., Kasemets K., Kurvet I., Milić M., Barbir R., Pem B., Erceg I. (2020). Impact of surface functionalization on the toxicity and antimicrobial effects of selenium nanoparticles considering different routes of entry. Food Chem. Toxicol..

[32] Shirazi A.N., Vadlapatla R., Koomer A., Yep K., Parang K. (2025). Selenium Nanoparticles as Versatile Delivery Tools. Pharmaceutics.

[33] Siddiqui S.A., Blinov A.V., Serov A.V., Gvozdenko A.A., Kravtsov A.A., Nagdalian A.A., Raffa V.V., Maglakelidze D.G., Blinova A.A., Kobina A.V. (2021). Effect of Selenium Nanoparticles on Germination of Hordéum Vulgáre Barley Seeds. Coatings.

[34] Zhang L., Xu Y., Wu D., Sun Y., Jiang X., Wei X. (2008). Effect of polyvinylpyrrolidone on the structure and laser damage resistance of sol–gel silica anti-reflective films. Opt. Laser Technol..

[35] Tański T., Matysiak W., Krzemiński Ł., Jarka P., Gołombek K. (2017). Optical properties of thin fibrous PVP/SiO_2_ composite mats prepared via the sol-gel and electrospinning methods. Appl. Surf. Sci..

[36] Szewczyk A., Prokopowicz M. (2020). Mesoporous silica pellets—A promising oral drug delivery system?. J. Drug Deliv. Sci. Technol..

[37] Bramanti E., Bonaccorsi L., Campanella B., Ferrari C., Malara A., Freni A. (2022). Structural characterization of electrospun tetraethyl ortosilicate (TEOS)/Polyvinylpyrrolidone (PVP) microfibers. Mater. Chem. Phys..

[38] Shar A.H., Lakhan M.N., Wang J., Ahmed M., Alali K.T., Ahmed R., Ali I., Dayo A.Q. (2019). Facile synthesis and characterization of selenium nanoparticles by the hydrothermal approach. Dig. J. Nanomater. Biostruct..

[39] Safo I.A., Werheid M., Dosche C., Oezaslan M. (2019). The role of polyvinylpyrrolidone (PVP) as a capping and structure-directing agent in the formation of Pt nano cubes. Nanoscale Adv..

[40] Pasieczna-Patkowska S., Cichy M., Flieger J. (2025). Application of Fourier Transform Infrared (FTIR) Spectroscopy in Characterization of Green Synthesized Nanoparticles. Molecules.

[41] Ahmadi S.M., Behnamghader A., Asefnejaad A. (2017). Sol-gel synthesis, characterization and in vitro evaluation of SiO_2_−CaO−P_2_O_5_ bioactive glass nanoparticles with various CaO/P_2_O_5_ ratios. Dig. J. Nanomater. Biostruct..

[42] Yu B., Zhou Y., Song M., Xue Y., Cai N., Luo X., Long S., Zhang H., Yu F. (2016). Synthesis of selenium nanoparticles with mesoporous silica drug-carrier shell for programmed responsive tumor targeted synergistic therapy. RSC Adv..

[43] Dang C., Liu M., Lin Z., Yan W. (2023). Selenium nanomaterials enabled flexible and wearable electronics. Chem. Synth..

[44] Piacenza E., Presentato A., Heyne B., Turner R.J. (2020). Tunable photoluminescence properties of selenium nanoparticles: Biogenic versus chemogenic synthesis. Nanophotonics.

[45] Kaur H., Chaudhary S., Kaur H., Chaudhary M., Jena K.C. (2022). Hydrolysis and Condensation of Tetraethyl Orthosilicate at the Air-Aqueous Interface: Implications for Silica Nanoparticle Formation. ACS Appl. Nano Mater..

[46] Malay O., Yilgor I., Menceloglu Y.Z. (2013). Effects of solvent on TEOS hydrolysis kinetics and silica particle size under basic conditions. J. Sol-Gel Sci. Technol..

[47] Serban B.A., Barrett-Catton E., Serban M.A. (2020). Tetraethyl Orthosilicate-Based Hydrogels for Drug Delivery—Effects of Their Nanoparticulate Structure on Release Properties. Gels.

[48] Raduly F.M., Raditoiu V., Raditoiu A., Grapin M., Constantin M., Răut I., Nicolae C.A., Frone A.N. (2024). Ag0–Ginger Nanocomposites Integrated into Natural Hydrogelated Matrices Used as Antimicrobial Delivery Systems Deposited on Cellulose Fabrics. Gels.

[49] Salah M., Elkabbany N.A.S., Partila A.M. (2024). Evaluation of the cytotoxicity and antibacterial activity of nano-selenium prepared via gamma irradiation against cancer cell lines and bacterial species. Sci. Rep..

[50] Indhira D., Aruna A., Manikandan K., Albeshr M.F., Alrefaei A.F., Vinayagam R., Kathirvel A., Priyan S.R., Kumar G.S., Srinivasan R. (2023). Antimicrobial and Photocatalytic Activities of Selenium Nanoparticles Synthesized from Elaeagnus indica Leaf Extract. Processes.

